# The last 25 years of research on bioflocculants for kaolin flocculation with recent trends and technical challenges for the future

**DOI:** 10.3389/fbioe.2022.1048755

**Published:** 2022-11-24

**Authors:** Markian S. Bahniuk, Fawad Alidina, Xiaoli Tan, Larry D. Unsworth

**Affiliations:** Department of Chemical and Materials Engineering, University of Alberta, Edmonton, AB, Canada

**Keywords:** Bacteria, bioflocculant, clay, polysaccharide, protein, tailings pond, wastewater

## Abstract

The generation of kaolin-containing wastewater is an inevitable consequence in a number of industries including mining, wastewater treatment, and bitumen processing. In some cases, the production of kaolin tailings waste during the production of bitumen or phosphate is as high as 3 times greater than the actual produced product. The existing inventory of nearly five billion barrels of oil sands tailings alone represents a massive storage and reclamation challenge, as well as a significant economic and environmental liability. Current reclamation options like inorganic coagulants and organic synthetic polymers may settle kaolin effectively, but may themselves pose an additional environmental hazard. Bioflocculants are an emerging alternative, given the inherent safety and biodegradability of their bio-based compositions. This review summarizes the different research attempts towards a better bioflocculant of kaolin, with a focus on the bioflocculant source, composition, and effective flocculating conditions. *Bacillus* bacteria were the most prevalent single species for bioflocculant production, with wastewater also hosting a large number of bioflocculant-producing microorganisms while serving as an inexpensive nutrient. Effective kaolin flocculation could be obtained over a broad range of pH values (1–12) and temperatures (5–95°C). Uronic acid and glutamic acid were predominant sugars and amino acids, respectively, in a number of effective bioflocculants, potentially due to their structural and charge similarities to effective synthetic polymers like polyacrylamide. Overall, these results demonstrate that bioflocculants can be produced from a wide range of microorganisms, can be composed of polysaccharides, protein or glycoproteins and can serve as effective treatment options for kaolin. In some cases, the next obstacle to their wide-spread application is scaling to industrially relevant volumes and their deployment strategies.

## 1 Introduction

The production of clay-containing wastewater tailings is an inevitable consequence of a wide variety of industries including mining and mineral processing (Wang et al., 2014; Shaikh et al., 2017; Liu et al., 2020). Four to six tons of phosphogypsum, phosphate tailings, are produced per ton of fertilizer: 100–280 million tons of phosphate tailings are estimated to be produced annually (Anagnostopoulas et al., 2022). Similarly, bitumen production from oil sands results in 3 times as much tailings as product, with a current daily production of ∼3 million barrels of tailings being added to the existing inventory of ∼4.8 billion barrels of mature fine tailings (Masliyah et al., 2011; Parajulee and Wania, 2014; Schindler, 2014). Tailings from phosphates, coal, and oil sands extraction are primarily kaolinite and are the impetus for kaolin-focused clay flocculation studies (Sabah et al., 2004; Ofori et al., 2011; Wang et al., 2014). Kaolin fine fluid tailings are particularly difficult to treat due to the fact that clay particles are highly charged and can remain colloidally stable for centuries, particularly as the particle size decreases (Hocking et al., 1999; Bergaya and Lagaly, 2006; Wang et al., 2014). Management of these tailings requires massive storage solutions, sequesters significant volumes of water along with the kaolin, and represents significant potential fiscal and environmental liabilities.

Coagulants and flocculants have been applied to try to solve the technical and environmental challenges posed by the management of tailings. Multiple flocculating mechanisms have been described for destabilizing colloidal clay dispersions: electrical double layer compression, charge neutralization and polymer bridging, sweep flocculation, and the electrostatic patch mechanism (Hocking et al., 1999; Shaikh et al., 2017). Furthermore, these flocculation mechanisms and the specific interactions between different types of flocculants and kaolin particles have both been extensively documented in established literature. (Hocking et al., 1999; Shaikh et al., 2017; Liu et al., 2020). While inorganic metal salt coagulants and synthetic polymers exhibit strong flocculating activity (FA) to settle suspended clays, there are additional complications associated with their application. Inorganic metal salts like ferric chloride, lime, alum, and polyaluminum chloride do not form strong flocs nor easily dewater and can be highly corrosive (Hocking et al., 1999; Pan et al., 1999; Salehizadeh and Shojaosadati, 2001; Salehizadeh and Shojaosadati, 2002; More et al., 2012; Busi et al., 2017). Polymers like polyacrylamide have been shown to have strong FA but multiple mechanisms for their degradation have been established, with iron, oxygen, or photolytic based free radical-mediated chain scission being the most relevant for tailings applications (Xiong et al., 2018). The environmental risk associated with monomeric acrylamide may be attenuated, however, given that acrylamide may also be further degraded by a range of soil and water microbes (Smith et al., 1997; Kay-Shoemake et al., 1998; Shukor et al., 2009; Labahn et al., 2010).

Bioflocculants are an emerging class of flocculant that have the potential to combine the efficacy of conventional synthetic polymer flocculants with the inherent biodegradability and long-term safety of bio-derived materials. Bioflocculants are composed of a range of biological building blocks: polysaccharides, proteins, nucleic acids, lipids, and combinations thereof (Yokoi et al., 1997; Zhao et al., 2013; Busi et al., 2017). Bioflocculants can be produced by a wide range of microorganisms including bacteria, fungi, and algae, typically as extracellular bio-polymeric substances, though there are studies characterizing intracellular and plant-based bioflocculants (Yim et al., 2007; Bala Subramanian et al., 2010; Liu et al., 2010; Pu et al., 2014; Choy et al., 2015). Current hurdles for the widespread adoption of bioflocculants include purported high production cost of purified flocculants and relatively low FA where

$$\begin{array}{c} FA=\frac{B-A}{A}\; \end{array}$$ (1)

and uses the control (B) and sample (A) optical density. (Kurane et al., 1986) Herein, the different research attempts from the last 25 years towards a better bioflocculant of kaolin are summarized. Flocculation parameters leading to an effective (FA ≥80%) settling of kaolin, including temperature, pH, metal-ion supplements, and bioflocculant concentration, are reported. Studies will be generally organized based on the bacterial genera producing the bioflocculant, or environment in which the bioflocculant-producing microorganism is found.

## 2 Bioflocculant-producing organisms and sources

### 2.1 Bacillus

*Bacillus* bacteria are the most frequently studied bioflocculant-producing microorganism. *Bacillus* are well-characterized, Gram-positive, aerobic or facultatively anaerobic, endospore-forming bacteria (Baron, 1996). *Bacillus* occur ubiquitously in soil, with species capable of existing in extreme environments including at high temperatures, salt concentrations, and pH conditions (Liu et al., 2014a; Robinson, 2014). *Bacillus* are well suited for bioflocculant production as they produce and excrete considerable amounts of enzymes, metabolites, and antibiotics and are employed in a host of industrial and pharmaceutical processes (Takami and Horikoshi, 2000).

The majority of the *Bacillus*-derived bioflocculants (Table 1) are primarily (>50%) polysaccharides, though there are 5 studies which examined protein bioflocculants. Of those, three were found to be polyglutamic acid, a known component of the *Bacillus* capsule (Lee et al., 2014; Lee et al., 2017; Ajayeoba et al., 2019). *Bacillus*-derived bioflocculants proved to be effective in an extremely wide pH range, with individual studies showing optimal performance at pH values between 1 and 12. This versatility allows for the use of these bioflocculants in industrial processes at acidic or alkaline conditions without requiring neutralization, streamlining treatment processes and costs.

**TABLE 1:** *Bacillus* genera and their bioflocculant performance.

Species name	Bioflocculant composition	Molecular weight (Da)	Measurement of flocculating activity	Mechanism	Optimal efficiency	Effective flocculation additives	Effective (≥80%) temperature range (°C) (max)	Effective (≥80%) pH range (max)	Effective (≥80%) concentration (max)	Optimal flocculating activity	References
*subtilis* MSBN17	58.6% S (4.2% NS, 41.3% UA, 1.2% AS) 17.8% P		0.2 mL culture supernatant, 5.0 mL-1% w/v CaCl_2_, 95 mL-5.0 g/L kaolin pH 8		94.26%	NaCl, MgCl_2_, K_2_H_2_PO_4_	0–60	>7, (10)	5 mg/L	pH 10	Sathiyanarayanan et al. (2013)
BS14	C:P ratio of 0.89		4 mL bioflocculant, 150 mg/L Ca^2+^ in 500 mL-5.0 g/L kaolin pH 7.5	Charge neutralization, Bridging	81.73%	Ca^2+^		5–7		1.12–2.70 mg bioflocculant/g S, 150 mg/L Ca^2+^	More et al. (2012)
*subtilis* DYU1	14.9% S, 2.7% UA, 4.4% P, 48.7% Glu	3.16–3.20E + 06	0.1 mL flocculant solution, 0.25 mL cation, 4.65 mL-5.0 g/L kaolin	Bridging, Charge neutralization	97%	>10 mM for Na^+^ or K^+^0.1–0.9 mM for Ca^2+^ or Mg^2+^<5.0 µM for Al^3+^ and Fe^3+^	30	5–8, (7)	40 mg/L	50 mM CaCO_3_40 mg-DYU500/L, 30°C, pH 7	Wu and Ye, (2007)
*licheniformis*	91.5% PS (16.4% UA), 7.1% pyruvic acid, 0.5% acetic acid, 8.4% P	6.89E + 04	5 mL-10% w/v CaCl_2_, 2.0 mL-culture broth, 993 mL -5.0 g/L kaolin pH 7.5	Charge neutralization	99.20%	9 mM Ca^2+^ 0.2 mM Al^3+^ 0.25 mM Fe^3+^	4–95, (30)	2–4 and 8–12	2 mg/L	30°C, 2 mg/L bioflocculant, 9 mM CaCl_2_	Ji et al. (2010)
*mojavensis* strain 32A	98.4% PS (28.7% sucrose, 29.85% maltose, 22.97% lactose), 1.6% P (38% Glu)		Bioflocculant, CaCl_2_, 5 g/L synthetic clay		89.7% at peak cost efficiency, 100% with 50 mL/L 3% CaCl_2_	5 mL/L of 3% CaCl_2_ for peak cost efficiency 50 mL/L for peak FA, Na^+^	25–75	1–7, (1)	≥3 mg/L, (≥50)	3 mg/L-bioflocculant, pH 1.0, 5 mL/L of 3% CaCl_2_ (cost efficiency) or 50 mL/L (max FA)	Elkady et al. (2011)
spp. UPMB13			50 mL-5.0 g/L kaolin pH 6.8, 0.5 mL culture broth 4.5 mL - 0.1% CaCl_2_	Charge neutralization, Bridging	94.20%	Na^+^, Ca^2+^, Mg^2+^		4–8	5 mL/L	CaCl_2_ 50 mL/L - 0.1% w/v 5 mL/L culture broth	Zulkeflee et al. (2012)
*circulans*	93.2% PS (19.8% UA, 6.5% PA, 0.7% CH_3_COOH) 6.4% P	4.85E + 04	5 mL-10%w/v CaCl_2_ 2.0 mL-culture broth 993 mL-200 mesh, 4.0 g/L kaolin, pH 7.5	Charge neutralization	99%	Al^3+^ (0.45 mM), Fe^3+^ (0.9 mM), or Ca^2+^ (9 mM)	15–95, (20)	4–10, (8–10)	2 mg/L	Bioflocculant 2 mg/L, 9 mM Ca^2+^	Li et al. (2009)
*licheniformis*	89% C (7.9:4:1 NS:AS:UA), 11% P	1.76E + 06	0.2 g/L CaCl_2_, 1 g/L bioflocculant. 40 mL-5 g/L kaolin	Adsorption, Bridging	98%	Ca^2+^, Mg^2+^, Na^+^, K^+^	10–85	5–12, (8)	4–32 mg/L	Bioflocculant 4–32 mg/L 0.2 g/L CaCl_2_	Wang et al. (2015)
*agaradhaerens* C9	65.42% PS, 4.70% P, 1.65% NA		0.1 mL bioflocculant, unspecified 5 g/L kaolin		95.29%	Cation independent	9–56, (29)	3.10–8.67, (6.53)	1.5 mg/L	1.5 mg/L bioflocculant, 29°C, pH 6.53	Liu et al. (2015a)
*pumilus* ZAP 028	75.4% C, 5.3% P, 15.4% UA		2 mL–culture supernatant, 3 mL–1% w/v CaCl_2_, 100 mL–4 g/L kaolin	Bridging	96.5% (purified), 89.7% (crude)	Ca^2+^, Mg^2+^, Mn^2+^, Al^3+^	≤100	Purified:3–11, (10) Crude: 7–9	0.1 mg/mL	0.1 mg/mL-bioflocculant, 1% w/v MnCl_2_, pH 10	Makapela et al. (2016)
*megaterium* SP1			2 mL culture broth, 5 mL-1% w/v CaCl_2_, 93 mL-5 g/L kaolin		94.32%		30	7		Culture medium 20 g/L glucose, 0.5 g/L beef extract, 30°C, pH 7	Luo et al. (2016)
*cereus* SK	57% P 43% C		0.2 mL bioflocculant (1–15 mg), 1 mL - 1 mg/L CaCl_2_ 100 mL-5 g/L kaolin pH 7.0		83.40%				1–15 mg/L, (12)	12 mg/L bioflocculant	Busi et al. (2017)
*mucilaginosus*	PS (19.1% UA, 47.4% NS, 2.7% AS)	2.60E+06	0.01 mL bioflocculant in culture broth 100 mL-5 g/L kaolin	Bridging	99.60%	Cation independent	<100		700 mg/L	0.1 mL bioflocculant in culture broth/L, 30°C, pH 8	Deng et al. (2003)
*licheniformis* P-104	Ultra-high molecular weight poly-γ-glutamic acid	1.24E + 06	5 mL–4.5 mM CaCl_2_, 5 mL bioflocculant, 190 mL–5 g/L kaolin	Charge neutralization	33.5 ± 1.6 OD-1 (purified), 46.66 ± 2.89 OD-1 (diluted culture broth)	Ca^2+^ (6 mM)		6	1.5 mg/L (purified), 10 mg/L crude γ-PGA in diluted culture broth	6 mM Ca^2+^, 1.5 mg L−1γ-PGA, pH 6.0 (purified bioflocculant), 0.5% v/v of 20-fold diluted culture broth (∼10 mg/L crude bioflocculant)	Zhao et al. (2013)
*licheniformis* CCRC 12826	<1% S, Polyglutamic acid	2.00E + 06	Various amounts of flocculant solution, 1 mL-90 mM CaCl_2_, 9.3 mL-5 g/L kaolin	Neutralization	8.5 1/OD	Ca^2+^ (13.5 mM), Fe^3+^, Al^3+^		7.0 (Ca^2+^), 6.4 (Fe^3+^), 7.1 (Al^3+^)	3.7 mg/L (purified), 13% crude culture broth	3.7 mg/L-bioflocculant, 13.5 mM Ca^2+^, pH 7.0	Shih et al. (2001)
sp. F19	66.4% PS (3.6%, w/w NS, 37.0%, w/w UA, 0.5% w/w AS), 16.4%, w/w P		Various amounts of bioflocculant and 5 g/L kaolin	Bridging	97%	Cation independent		2–7, (2)	1–20 mg/L, (2)	2 mg/L, pH 3.0	Zheng et al. (2008)
*megaterium* TF10	76.9%, w/w PS (93.6% w/w NS), 23.0% w/w P	1–2.5E + 03	bioflocculant, 5.6 mM CaCl_2_, 80 mL-4 g/L kaolin	Bridging	95.5 ± 1.0%	5.6 mM CaCl_2_	<100		30.2 mg/L	30.2 mg/L-bioflocculant 5.6 mM CaCl_2_	Yuan et al. (2011)
*subtilis* R 23	Poly γ-glutamic acid	6.20E + 06	Various bioflocculant, 4.5 mM CaCl_2_, 9.3 mL-5 g/L kaolin	Neutralization, Bridging	30.32 ± 1.4 1/OD	Ca^2+^ (8–10 mM), Mg^2+^ (1.0 mM), Al^3+^ (0.1 mM), Fe^3+^ (0.1 mM)		5–8, (7.5)	7.5 mg/L, ≤10 mg/L	7.5 mg/L -bioflocculant, 8.0 mM Ca^2+^, pH 7.5	Bajaj and Singhal, (2011)
sp. MAYA, sp. Gilbert, *Cobetia* sp. OAUIFE	43.2% P, 9.0% UA, 0.53% NS		2 mL bioflocculant, 3 mL-1% CaCl_2_, 100 mL kaolin	Adsorption	96%	Li^+^, Na^+^, Ca^2+^, Mg^2+^, Mn^2+^, Al^3+^		3–9, 11–12, (12)	1.0 mg/mL	1.0 mg/mL-bioflocculant, pH 12	Ugbenyen et al. (2015)
*licheniformis*	167 μg/mL PS, 48 μg/mL P		1–7 mL 5 mg/mL bioflocculant, 3 mL 10 g/L–CaCl_2_, 100 mL-10 g/L kaolin pH 7–7.2	Particle-particle interbridging, Neutralization	96%	CaCl_2_		3–9, (7)	0.32 mg/mL	0.32mg/mL-bioflocculant, pH 7	Karthiga Devi and Natarajan, (2015)
*firmus*	90 μg/mL PS, 30 μg/mL P		1–7 mL 5 mg/mL bioflocculant, 3 mL 10 g/L - CaCl_2_, 100 mL-10 g/L kaolin pH 7–7.2	Particle-particle interbridging, Neutralization	92%	CaCl_2_		5–10, (9)	0.28 mg/mL	0.28 mg/mL-bioflocculant, pH 9	Karthiga Devi and Natarajan, (2015)
*aryabhattai* PSK1	PS (76.67% glucose, 23.24% rhamnose) P (17.9% Arg, 16.7% Phe, 14.2% Thr, 10.8% Tyr)		Various amounts of bioflocculant, CaCl_2_, 5 g/L kaolin	Bridging, Neutralization	94.56%	Na^+^, K^+^, Ca^2+^, Mg^2+^	5–80, (50)	2–9, (2)		5–9 g/L kaolin pH 2, 50°C, Ca^2+^	Abd El-Salam et al. (2017)
*agaradhaerens* C9	74.12% PS, 4.51% P	1.37E + 05	100 μL bioflocculant, 1 mL of 1% CaCl_2_, 60 mL-5 g/L kaolin		92.67%				12.94 g/L	24 h conversion of rice bran to bioflocculant	Liu et al. (2017)
*marisflavi* NA8	74% PS, 25% P, 1% NA		3 mL culture supernatant, 3 mL 1% w/v CaCl_2_, 100 mL-5 g/L kaolin	Neutralization	85%	Fe^3+^, Mg^2+^, Na^+^, Ca^2+^, Cu^2+^	10–100	2–8, (6)		Fe^3+^, pH 6	Bukhari et al. (2020)
*subtilis*	91% C, 5.1% P		5 g/L kaolin pH 7.0, 1.5 mL of 10% CaCl_2_ 10 mL of bioflocculant liquor		>99%	Ethanol		4–5		Synthetic medium enriched with 1,100 mg/L ethanol	You et al. (2008)
strain BS9	0.4–7% P, 4–7% C		0.5 mg/L-5.0 g/L bioflocculant, 9 mL-5 g/L kaolin with and without 100 mg/L CaCl_2_	Bridging, Adsorption	81.7%	Ca^2+^			3 g/L slime EPS	3 g/L slime EPS, 100 mg/L CaCl_2_	Bala Subramanian et al. (2010)
*sphaericus and Rhizobium radiobacter*			10 mL fermentation liquor, 1.5 mL 10% CaCl_2_, 1L-5 g/L kaolin pH 7.0		97.35%					100-day fermentation with a 5:1 ratio of synthetic medium and fermentation liquor	Zhao et al. (2012)
*licheniformis (KX657843) isolated from Earthworm*			2 mg/L–20 mg/L bioflocculant, 0.2 M 1% w/v CaCl_2_, 5 g/L kaolin	Monolayer adsorption, Bridging	83%	CaCl_2_		6–11, (11)	4 mg/L (only 74%)	pH 11, 4 mg/L EPS, 2% CaCl_2_	Biswas et al. (2020)
*firmus (NCIM 2264)*			5 g/L (1–7 mL) bioflocculant, 3 mL of 10 g/L CaCl_2_, 10 g/L kaolin	Bridging	92%	CaCl_2_		3, 5, 7, 9, 10 (9)	5 g/L, 6 mL	pH 9, 5 g/L 6 mL bioflocculant, 10 g/L CaCl_2_	Devi and zand Natarajan, (2015)
*cereus* AKVCRR01			1 mL bacterial supernatant, 3 mL of 1% CaCl_2_, 9 mL of 5.0 g/L kaolin, 2000 ppm K_2_CrO_4_		77.50%	CaCl_2_, K_2_CrO_4_			1 mL bacterial supernatant	1 mL bacterial supernatant, 3 mL of 1% CaCl_2_, 9 mL of 5.0 g/L kaolin, 2000 ppm K_2_CrO_4_	Monga et al. (2022)
AKVCRR03			1 mL bacterial supernatant, 3 mL of 1% CaCl_2_, 9 mL of 5.0 g/L kaolin, 2000 ppm K_2_CrO_4_		80.15%	CaCl_2_, K_2_CrO_4_			1 mL bacterial supernatant	1 mL bacterial supernatant, 3 mL of 1% CaCl_2_, 9 mL of 5.0 g/L kaolin, 2000 ppm K_2_CrO_4_	Monga et al. (2022)
AKVCRR04			1 mL bacterial supernatant, 3 mL of 1% CaCl_2_, 9 mL of 5.0 g/L kaolin, 2000 ppm K_2_CrO_4_		89.75%	CaCl_2_, K_2_CrO_4_			1 mL bacterial supernatant	1 mL bacterial supernatant, 3 mL of 1% CaCl_2_, 9 mL of 5.0 g/L kaolin, 2000 ppm K_2_CrO_4_	Monga et al. (2022)
AKVCRR05			1 mL bacterial supernatant, 3 mL of 1% CaCl_2_, 9 mL of 5.0 g/L kaolin, 2000 ppm K_2_CrO_4_		89.88%	CaCl_2_, K_2_CrO_4_			1 mL bacterial supernatant	1 mL bacterial supernatant, 3 mL of 1% CaCl_2_, 9 mL of 5.0 g/L kaolin, 2000 ppm K_2_CrO_4_	Monga et al. (2022)
AKVCRR06			1 mL bacterial supernatant, 3 mL of 1% CaCl_2_, 9 mL of 5.0 g/L kaolin, 2000 ppm K_2_CrO_4_		80.34%	CaCl_2_, K_2_CrO_4_			1 mL bacterial supernatant	1 mL bacterial supernatant, 3 mL of 1% CaCl_2_, 9 mL of 5.0 g/L kaolin, 2000 ppm K_2_CrO_4_	Monga et al. (2022)

S, sugar; NS, neutral sugar; UA, uronic acid; AS, amino sugar; P, protein; C, carbohydrate; PS, polysaccharide; Glu, glutamic acid; Arg, arginine; Phe, phenylalanine; Thr, threonine; Tyr, tyrosine; NA, nucleic acid.

In addition to their general acceptance in industrial processes, these bioflocculants exhibit a very high flocculating activity (FA). For instance, *Bacillus mucilaginosus* produced a bioflocculant with 99.6% FA using 0.1 mL culture broth/L at pH 8 and 30°C, while retaining good FA at temperatures as high as 100°C (Deng et al., 2003). *Bacillus circulans* produced a bioflocculant with an optimal FA of 99% using 2 mg/L bioflocculant and 9 mM Ca^2+^, while retaining good FA at temperatures as high as 95°C (Li et al., 2009) *Bacillus subtilis* produced a bioflocculant capable of 99.7% FA using a synthetic medium enriched with 1,100 mg/L ethanol (You et al., 2008).

### 2.2 Pseudomonas

*Pseudomonas* are rod-shaped, aerobic Gram-negative bacteria with a polar flagella for motility that are also commonly used for bioflocculant production (Madigan et al., 2008). As a result of their metabolic versatility, they can be found in soil, water and vegetation (Baron, 1996). This versatility allows for the use of a variety of organic compounds as nutrients, as well as survival in a broad range of temperatures and salts and even in the presence of some weak antiseptics and antibiotics (Baron, 1996). *Pseudomonas* bacteria are known to produce biofilms by excreting polysaccharides, a quality which is directly applicable for bioflocculation (Hassett et al., 2002).

The studies on *Pseudomonas*-derived bioflocculants (Table 2) all used kaolin test solutions of 4–5 g/L, allowing straightforward comparisons with *Bacillus* results. Calcium was the most commonly used cation, though two studies showed that monovalent cations could also be used for effective flocculation (Gomaa, 2012; Farag et al., 2014). As with *Bacillus* bioflocculants, polysaccharides were the most prominent structural element, though most were composed of some protein as well. Of note is the bioflocculant produced by *Pseudomonas* Sp. HP2 because of the large proportion of proline, glutamic, and aspartic acids found in the considerable proteinaceous component (Qi et al., 2019). Glutamic and aspartic acid sidechains differ by a single methylene group, and polyglutamic acids were found to be effective bioflocculants produced by *Bacillus* bacteria. The frequency of proline (25.5% of all amino acids) is very high and may influence the protein secondary structure away from conventional α-helices and β-sheets and toward intrinsically disordered structures and/or polyproline helices (Morgan and Rubenstein, 2013; Bahniuk et al., 2020). Three of the *Pseudomonas* studies attempt to address the economic concerns associated with bioflocculant production by utilizing alternative nutrient sources, driving down production costs while maintaining FA >90% (Liu et al., 2016; Guo et al., 2018a; Qi et al., 2019).

**TABLE 2:** *Pseudomonas* genera and their bioflocculation performance.

Species name	Bioflocculant composition	Molecular weight (Da)	Measurement of flocculating activity	Mechanism	Optimal efficiency	Effective flocculation additives	Effective (≥80%) temperature range (°C) (max)>	Effective (≥80%) pH range (max)	Effective (≥80%) concentration (max)	Condition for optimal flocculating activity	References
Sp. HP2	38.49% P (26.5% Pro, 10.9% Asp, 7.9% Glu), 47.83% MS (33.3% rhamnose, 24.8% glucose, 20.2% mannose)		100 μL cell supernatant 1.0 mL-1.0% (w/v) CaCl_2_, 40 mL-0.4% (w/v) kaolin	Adsorption	92.50%		Room temperature			0.5% rice straw as sole carbon source	Qi et al. (2019)
*boreopolis* G22	66.3% PS, 10.4% P, 12.6% NA	3.98E + 06	200 μL sample, 1.0 mL-10% (w/v) CaCl_2_ 40 mL-0.5% (w/v) kaolin		97.1%		30–80	4–11	3.5 mg/L	Fermentation: 96 h, 30°C, 1% inoculum, 1% biomass	Guo et al. (2018a)
*veronii* L918	77.14% PS, 4.84% P	2.48E + 04	100 μL fermentation liquor, 1 mL-1% CaCl_2_, 60 mL-5 g/L kaolin		91.93%		28–32	5.0, 5.5, 6.5–8.0		300 mL/L Peanut hull hydrolysate carbon source, yeast/casein/tryptone nitrogen source, 24 h growth	Liu et al. (2016)
*aeruginosa* ATCC-10145	89% C: 30.6% NS, 2.35% UA, 0.78% AS; 27% P		0.5 mL culture supernatant, 4.5 mL-1% CaCl_2_ 100 mL-5 g/L kaolin	Bridging	96.16%	Ca^2+^, Zn^2+^, Mg^2+^, Cu^2+^, Na^+^, K^+^	30–40	7	1%	1% bioflocculant, 1 mM Na+, 35°C, pH 7.0	Gomaa, (2012)
Sp. 38A	94.61% C (95.5% glucose), 2.85% P (60% Glu)		0.5 mL culture supernatant, 10 mL-3% (w/v) CaCl_2_ 200 mL-5 g/L kaolin pH 7	Charge neutralization	99.89%	Ca^2+^, Na^+^, K^+^	5–25	7	4–45 mg/L	15 mg/L bioflocculant, pH 7, 0.14% w/v CaCl_2_	Farag et al. (2014)
Strain BS2	0.4–7% P, 4–7% C		0.5 µg/mL-5.0 mg/mL bioflocculant, 9 mL-5 g/L kaolin with and without 100 mg/L CaCl_2_	Bridging, Adsorption	81.0–82.3%	Ca^2+^			2–3 g/L slime EPS	3 g/L slime EPS, 100 mg/L CaCl_2_	Bala Subramanian et al. (2010)
*aeruginosa* AKVCRR02			1 mL bacterial supernatant, 3 mL of 1% CaCl_2_, 9 mL of 5.0 g/L kaolin, 2000 ppm K_2_CrO_4_		77.50%	CaCl_2_, K_2_CrO_4_			1 mL bacterial supernatant	1 mL bacterial supernatant, 3 mL of 1% CaCl_2_, 9 mL of 5.0 g/L kaolin, 2000 ppm K_2_CrO_4_	Monga et al. (2022)

P, protein; Pro, proline; Asp, aspartic acid; Glu, glutamic acid; MS, monosaccharide; PS, polysaccharide; NA, nucleic acid; C, carbohydrate; NS, neutral sugar; UA, uronic acid; AS, amino sugar.

*Pseudomonas boreopolis* G22 produced a bioflocculant capable of an optimal FA of 97.1% at 60°C after 4 days of fermentation using wood dust as an inexpensive carbon source (Guo et al., 2018a). *Pseudomonas* Sp. 38A produced a bioflocculant with an optimal FA of 99.89% at pH 7.0 when 15 mg/L bioflocculant and 0.14% w/v CaCl_2_ were used (Farag et al., 2014). *Pseudomonas aeruginosa* produced a bioflocculant with an optimal FA of 96.16% when monovalent Na^+^ ions were included (Eman Zakaria, 2012).

### 2.3 Wastewater bacteria

Wastewater contains a diverse population of bacteria that can serve as a source for the discovery of novel bacteria capable of efficient bioflocculation, as well as an inexpensive and ubiquitous source of nutrition for bioflocculant-producing bacterial cultures and a host of potential pollutants including suspended wastewater solids, estrogen, arsinite, dyes, cadmium and other heavy metals (Liu et al., 2009; Zhong et al., 2014a; Cao et al., 2015; Chen et al., 2016). The use of kaolin to screen and initially assess bioflocculant performance is warranted (Table 3), given the zeta potential values for wastewater suspended solids is comparable to that of kaolin at ∼ -32 mV (Nguyen et al., 2016). Table 3 summarizes the physical characteristics and flocculation performance of bioflocculants produced by wastewater-derived bacteria.

**TABLE 3:** Bacterium from wastewater and their flocculation performance.

Wastewater source/bacterium	Bioflocculant composition	Molecular weight (Da)	Measurement of flocculating activity	Mechanism	Optimal efficiency	Effective flocculation additives	Effective (≥80%) temperature range (°C) (max)	Effective (≥80%) pH range (max)	Effective (≥80%) concentration (max)	Condition for optimal flocculating activity	Reference
Corn ethanol Wastewater/*Klebsiella variicola* B16	81.8% PS (28.92% NS, 4.04% AS, 11.69% UA), 15.9% P		1 mL-10 g/L CaCl_2_, 0.25 mL bioflocculant, 20 mL-5 g/L kaolin	Neutralization, Bridging	96.18%	Ca^2+^, Mn^2+^, Mg^2+^, Fe^2+^	20–100, (60)	3–12, (9–10)	3.53–23.53 mg/L, (5.88)	5.88 mg/L, 60°C, pH 9–10, 1 mL-10 g/L CaCl_2_	Xia et al. (2018)
Potato Starch Wastewater/*Rhodococcus erythropolis*	92.3% sugar (49.7% NS, 13.2% UA, 12.4% AS) 7.6% P	3.85E + 05	100 mL-4 g/L pH 7.5 kaolin, 50 mg CaCl_2_, 2.0 mg bioflocculant		95.6%		10–70	6.5–8.5, (7.5)	1.18 g/L	1.18 g/L, pH 7.5	Guo et al. (2018b)
Amino-8-naphthol-3,6-disulfonic acid wastewater sludge/*Klebsiella pneumoniae*	82.4% PS (5:2:4 NS:AS:UA), 14.2% P	2.00E + 06	2–100 mg/L bioflocculant, 5 mL-9 mM CaCl_2_, bioflocculant, 993 mL-4 g/L kaolin, pH 7.0	Neutralization, Adsorption, Bridging	95.1%	Cation independent	20–70, (30)	2–7, (5)	20–180 mg/L, (40)	40 mg/L bioflocculant, 30°C, pH 5	Zhong et al. (2014b)
Chromatropic acid wastewater/*Sphingomonas yabuuchiae*	91% PS (4:2:3 NS:AS:UA), 9% P	1.90E + 06	2–100 mg/L bioflocculant, 5 mL-9 mM CaCl_2_, bioflocculant, 993 mL-4 g/L kaolin, pH 7.0	Bridging, Neutralization	92.5%	Cation independent	10–50, (30)	2–7, (5)	5–70 mg/L, (20)	20 mg/L bioflocculant, 30°C, pH 5	Zhong et al. (2014a)
Methanol Wastewater/*Turicibacter sanguinis*	74.1% PS (3:4:3 NS:AS:UA), 24.2% P	1.90E + 06	1 mL of fermentation broth in 100 mL-4 g/L kaolin		95.70%						Cao et al. (2015)
Cassava wastewater biological aerated filter sludge	PS, P	∼45% PS, ∼23% P	1.0–2.0 mL culture broth, 100 mL-5 g/L kaolin		92.67%		10–30, (10)	5–7, (7)	6–9 mg/L, (6)	6 mg/L bioflocculant, 10°C, pH 5.0	Liu et al. (2009)
Paper mill wastewater activated sludge/*Klebsiella* sp. ZZ-3	84.6% PS, 6.1% P	87.1% 2.81E+05, 12.7% 1.97E+06	1 mL bioflocculant, 50 mL-4 g/L kaolin	Bridging	93.90%	Cation independent	<115	3–7, 9, (5)	1.3–5.0 mg/L, (5.0)	5 mg/L bioflocculant, pH 5.0	Yin et al. (2014)
Municipal wastewater treatment plant activated sludge/*Enterobacter* sp.ETH-2	91.7% PS (1:2.9:9.8 mannose, glucose, galactose), 1.8% P	6.03E+05–1.82E+06	1 mL bioflocculant, 50 mL-4 g/L kaolin	Bridging	94%	Cation independent	40–100, (40)	3–9, (5)	0.14–66 mg/L, (1.3)	1.3 mg/L bioflocculant pH 5.0, 40°C	Tang et al. (2014)
Potato starch wastewater/*Paenibacillus polymyxa*	96.2% S	1.16E+06	2.0 mg bioflocculant, 50 mg CaCl_2_, 100 mL-4 g/L kaolin, pH 7.5	Neutralization, Bridging	95.60%	Ca^2+^		7.5–9.5, (8.5)	20 mg/L	20 mg/L bioflocculant and 0.5 g/L Ca^2+^, pH 8.5	Guo et al. (2015)
Phenol-containing wastewater treatment plant sludge/*Stenotrophomonas maltophilia*	71.2% PS (8.8% AS, 2.9% NS 59.3%UA) 27.9% P	1.50E+06	0.5 mL bioflocculant, 4.5 mL-1% CaCl_2_, 45 mL-5 g/L kaolin	Bridging	95.29%		30	6		800 mg/L phenol, 2% dissolved oxygen, 10% inoculum, pH 6.0, 30°C	Chen et al. (2016)
Activated sludge/*Ochrobactium ciceri W2*	PS, P		10 mL fermentation liquor, 1.5 mL-10% w/v Ca(OH)_2_, 1L-5 g/L kaolin pH 7.5		>92%	Ca^2+^	30–80, (30)	0–10, (6)		30°C, pH 6.0	Wang et al. (2013)
Seafood processing plant activated sludge/*Enterobacter cloacae* WD7	29.4% NS, 14.2% UA, 0.93% AS		0.1 mL sample, 0.9 mL-1% CaCl_2_, 9 mL-5 g/L kaolin pH 7.0	Neutralization	≥91%	Ca^2+^, Fe^3+^	4–50, (50)	2–8, (6)	1–2.5 mg/L, (2)	2 mg/L bioflocculant, 40 mM CaCl_2_	Prasertsan et al. (2006)
Biological sludge	76.3% S, 4.21% P		2 mL crude bioflocculant in 300 mL-4 g/L kaolin	Neutralization	99.50%		5–80, (5)	5–11, (11)	0.33–3.33% (v/v), (3.00%)	pH 10.5, 3.0% v/v bioflocculant	Sun et al. (2012)
*Chryseobacterium daeguense W6*	32.4% P, 13.1% S, 6.8% NA		0.1 mL bioflocculant added to unspecified volume of 4 g/L kaolin	Bridging	96.90%	Best with no added cations	0–65, (15)	4.0–9.1, (5.6)	0.3–8.2 mg/L, (1.2)	1.2 mg/L bioflocculant pH 5.6, 15°C	Liu et al. (2010)
Propylene oxide wastewater activated sludge/*Agrobacterium* M-503 (crude)	85.9% NS, 9.2% UA 2.1% AS, 3.0% P	8.1E+04	0.05 mL bioflocculant, 0.9 mL-10 g/L CaCl_2_, 10 mL-5 g/L kaolin pH 7.0		917 U*		30–121	8–12		pH 10, 70°C	Li et al. (2010)
Propylene oxide wastewater activated sludge/*Agrobacterium* M-503 (purified)	85.9% NS, 9.2% UA 2.1% AS, 3.0% P	8.1E+04	0.05 mL bioflocculant, 0.9 mL-10 g/L CaCl_2_, 10 mL-5 g/L kaolin pH 7.0		916 U*		20–121	8–12		pH 11, 50°C	Li et al. (2010)
Palm oil effluent/*Staphylococcus cohnii* ssp			0.2 mL bioflocculant, 2 mL-1% w/v CaCl_2_, 100 mL-5 g/L kaolin pH 7.0	Bridging, Neutralization	88.90%	Al^3+^	40	6–8, (7)	1.2 mg/L	1.2 mg/L	Wong et al. (2012)
Municipal wastewater activated sludge/*Klebsiella* sp	100% PS	8.0E+05	2 mL culture broth, 5 mL-1% CaCl_2_, 100 mL-5 g/L kaolin	Bridging, Neutralization	86.50%				0.034 g/L	0.034 g/L after 60 h cultivation	Yang et al. (2012)
Municipal wastewater sludge/*Serratia* strain BS8	0.4–7% P, 4–7% C		0.5 µg/mL-5.0 mg/mL bioflocculant, 9 mL-5 g/L kaolin with and without 100 mg/L CaCl_2_	Bridging, Adsorption	81.4%	Ca^2+^			3 g/L slime EPS	3 g/L slime EPS, 100 mg/L CaCl_2_	Bala Subramanian et al. (2010)
Municipal wastewater sludge/*Yersinia* strain BS11	0.4–7% P, 4–7% C		0.5 µg/mL-5.0 mg/mL bioflocculant, 9 mL-5 g/L kaolin with and without 100 mg/L CaCl_2_	Bridging, Adsorption	80.9–85.7%	Ca^2+^			2–5 g/L slime EPS	3 g/L slime EPS, 100 mg/L CaCl_2_	Bala Subramanian et al. (2010)
Municipal wastewater sludge/*Microbacterium* strain BS15	0.4–7% P, 4–7% C		0.5 µg/mL-5.0 mg/mL bioflocculant, 9 mL-5 g/L kaolin with and without 100 mg/L CaCl_2_	Bridging, Adsorption	81.2–84.4%	Ca^2+^			2–3 g/L slime EPS	3 g/L slime EPS, 100 mg/L CaCl_2_	Bala Subramanian et al. (2010)
Municipal sewage treatment activated sludge/*Rhodococcus erythropolis*	99.2% P (16.7% Ala, 15.2% Glu 11.8% Asp)	4.21E + 05	2.0 mg bioflocculant, 50 mg CaCl_2_, 100 mL-4 g/L kaolin pH 7.5	Bridging, Neutralization	94.5% (broth), 92.8% (slime)	Ca^2+^	≤60		18–28 mg/L, max at 22 mg/L	20 mg/L bioflocculant produced in alkaline thermal treated sludge with 25 g/L suspended solids, 0.5 mg/mL Ca^2+^	Guo et al. (2014)
Municipal sewage treatment activated sludge/*Rhodococcus erythropolis*	84.6% P (18.5% Ala, 13.3% Glu, 10.3% Asp), 15.2% S	3.99E + 05	2.0 mg bioflocculant, 15 mg CaCl_2_, 100 mL-4 g/L kaolin pH 7.5	Bridging, Neutralization	90.8%		<60			2.0 mg of fermented broth	Guo et al. (2013)
Wastewater Sludge/*Cloacibacterium Normanense* NK6	12.7% C, 21.8% P		0.25–4.0 mL bioflocculant, 150 mg/L Ca^2+^ in 5 g/L kaolin pH 7	Bridging, Adsorption	86.8%	Ca^2+^			1–3 mg broth EPS/g kaolin	2.6 mg broth EPS/g kaolin, 150 mg/L Ca^2+^	Nouha, (2016)
Wastewater Sludge/*Cloacibacterium Normanense* NK6	12.7% C, 21.8% P		0.25–4.0 mL bioflocculant, 150 mg/l Ca^2+^ in 5 g/L kaolin pH 7	Bridging, Adsorption	94.20%	Ca^2+^			1–3 mg slime EPS/g kaolin	1.3 mg slime EPS/g kaolin, 150 mg/L Ca^2+^	Nouha, (2016)
Bioflocculants of raw sludge extracted by 5 g/L of EDTA	0.9 g/g P, 0.18 g/g C 0.48 mg/g NA extracted ≥5.0 g EDTA/L of sludge		Bioflocculant, 150 mg Ca^2+^/L, 5 g/L kaolin pH 7.5		94%	Ca^2+^			∼1.7–4.0 mg/g kaolin	2.4 mg/g kaolin, extracted with 5.0 g EDTA/L of sludge, 150 mg/L Ca^2+^	Nguyen et al. (2016)
Rice Stover/*Rhodococcus erythropolis*	Polysaccharides 95.6% (w/w)	3.93E+05	2.0 mg bioflocculant, 50 mg CaCl_2_, 100 mL-4 g/L kaolin, pH 7.5		94.10%	Ca^2+^	10–90	6.5–9.5, (7.5)	20 mg/L	20 mg/L bioflocculant, 30°C, pH 7.5	Guo et al. (2017)
Defatted olive mill wastewater	P, PS, UA, Phenol, Tannin		0–160 mg/L bioflocculant, 1 g/L kaolin	Bridging, Sweeping	93%			3–12, (11)	60 mg/L	60 mg/L bioflocculant, pH 11	Bouaouine et al. (2020)

PS, polysaccharide; NS, neutral sugar; UA, uronic acid; AS, amino sugar; P, protein; S, sugar; Ala, alanine; Glu, glutamic acid; Asp, aspartic acid; C, carbohydrate; NA, nucleic acid.

A wide range of wastewater bacteria were studied, with *Rhodococcus* producing four bioflocculants (Guo et al., 2013; Guo et al., 2014; Guo et al., 2017; Guo et al., 2018b). Most of the bioflocculants summarized in the table performed well at relatively low concentrations. The majority were stable up to 60°C due to being primarily polysaccharide-based, though some were found to be primarily composed of protein (Liu et al., 2010; Guo et al., 2013; Guo et al., 2014) or lipids and/or nucleic acids (Bala Subramanian et al., 2010; Nouha, 2016). The localization of bioflocculant molecules was examined, in some instances, by quantifying the different FA of slime and capsular culture fractions to determine whether the bioflocculants themselves were extracellular or cell-bound, respectively, or identified as being an intracellular protein (Bala Subramanian et al., 2010; Liu et al., 2010; Guo et al., 2013; Guo et al., 2014; Nouha, 2016).

Some of the most efficient bioflocculation results came from the use of a biological sludge-derived bioflocculant, yielding an FA of 99.5% when the suspension was maintained at pH 10.5 and dosed with a concentration of bioflocculant at 3.0% v/v (Sun et al., 2012). *Chryseobacterium daeguense* W6 produced a bioflocculant with an optimal FA of 96.9% by maintaining the suspension at pH 5.6 and 15°C, and then dosed with 1.2 mg/L bioflocculant (Liu et al., 2010). Finally, corn ethanol wastewater was a low cost substrate to produce a bioflocculant with optimal FA of 96.18%, when suspension was maintained at pH 8.1 and 30°C (Xia et al., 2018).

### 2.4 Plant-derived bioflocculants

Bacteria are not the only source of bioflocculating materials. Plants can be employed directly as bioflocculants (Table 4), in addition to serving as nutrients for bacteria. Ancient African, Indian, and Chinese cultures were documented practicing plant-mediated bioflocculation ∼2000 years ago, particularly for the purification of drinking water (Asrafuzzaman et al., 2011; Choy et al., 2015). The opportunity to use readily available, local florae is advantageous relative to bacteria in that no significant manufacturing infrastructure, beyond farmland, is necessary to produce plant-based bioflocculants. The availability of these materials for water purification is critical in rural locations, and municipalities where local infrastructure is lacking or overwhelmed.

**TABLE 4:** Plant-derived bioflocculants.

Plant bioflocculants	Bioflocculant composition	Molecular weight (Da)	Measurement of flocculating activity	Mechanism	Optimal efficiency	Effective flocculation additives	Effective (≥80%) temperature range (°C) (max)	Effective (≥80%) pH range (max)	Effective (≥80%) concentration (max)	Condition for optimal flocculating activity	References
Coccinia indica fruit	20% P, 26.7% F, 51.7% C, 1.7% L Lim (2012)		10–100 NTU kaolin solution		94%			7.5	0.4 mg/L	0.4 mg/L, pH 7.5, 100 NTU kaolin	Patale and Pandya (2012), Choy et al. (2015)
Moringa oleifera			25–120 NTU clay solutions		94.1		26–32		100 mg/L	100 mg/L bioflocculant, 100 NTU clay	Asrafuzzaman et al. (2011); Choy et al. (2015)
Cicer arietinum seeds	18.2% P, 16.4% F, 57.3% C, 5.7% L Ghadge et al. (2008); Lim (2012)		25–120 NTU clay solution		95.89%		26–32		100 mg/L	100 mg/L bioflocculant, 95 NTU clay	Asrafuzzaman et al. (2011); Choy et al. (2015)
Dolichos lablab			25–120 NTU clay solution		88.9%		26–32		100 mg/L	100 mg/L bioflocculant, 100 NTU clay	Asrafuzzaman et al. (2011); Choy et al. (2015)
Ceratonia siliqua			17.5, 35 and 70 NTU turbid kaolin water		100%			10	20 mg/L	20 mg/L bioflocculant, pH 10, 17.5 NTU kaolin	Sciban et al. (2005); Choy et al. (2015)
Phaseolus vulgaris seeds	22.4% P, 24.0% F, 52% C, 1.7% L Brink et al. (2006)		17.5, 35 and 70 NTU turbid kaolin water		80%			10–11	5 mg/L	5 mg/L bioflocculant, pH 10, 17.5 NTU kaolin	Sciban et al. (2005); Choy et al. (2015)
Vigna unguiculata	23.8% P, 10.8% F, 60.9% C, 1.3% L Yalçın (2007); Lim (2012)	∼6 kDa	450–550 NTU turbid kaolin water pH 7.3		86% (Crude) 92% (purified)			7.5	12.5 μg/mL (crude) 2 μg/mL (purified)	Crude: 12.5 μg/mL bioflocculant 2 h settling Purified: 0.6 M NaCl fraction, 2 μg/mL, 2 h settling	Marobhe et al. (2007); Choy et al. (2015)
*Parkinsonia aculeate*		∼6 kDa	450–550 NTU turbid kaolin water pH 7.3		86% (Crude) 92% (purified)			7.5	7.5 μg/mL (crude) 1.5 μg/mL (purified)	Crude: 7.5 μg/mL bioflocculant, 2 h settling Purified: 0.6 M NaCl fraction, 1.5 μg/mL, 2 h settling	Marobhe et al. (2007); Choy et al. (2015)
Pectin	Polysaccharide (primarily galacturonic acid)		0.1 mL pectin, 0.25 mL cation, 4.65 mL-5 g/L kaolin	Neutralization, Bridging	∼94%	0.2 mM Al^3+^, 0.2 mM Fe^3+^	30	3	10–40 mg/L, max 30 mg/L	30 mg/L pectin, 0.2 mM Al^3+^ or Fe^3+^, pH 3	Yokoi et al. (2002)
Mallow mucilage			>25 mg/L bioflocculant, 10 mg/L humic acid, 100 mg/L kaolin		96.3–97.4%	0.025 mM Al^3+^	22	5.9	10–50 mg/L	12 mg/L bioflocculant, 20 min sedimentation, pH 5.9, 22°C	Anastasakis et al. (2009)
Okra mucilage			>50 mg/L bioflocculant, 10 mg/L humic acid, 100 mg/L kaolin		93–97.3%	0.025 mM Al^3+^	22	5.9	3–25 mg/L	5 mg/L bioflocculant, 30 min sedimentation, pH 5.9, 22°C	Anastasakis et al. (2009)

P, protein; F, fiber; C, carbohydrate; L, lipid.

The studies summarized herein focus more on preliminary confirmation of bioflocculation rather than FA optimization or purification of specific entities from the bulk plant material. Additionally, some of these studies utilized various concentrations of suspended clays, based upon nephelometric turbidity units (NTUs) rather than weight percentage; some instances of optimal activity depend upon the initial water NTUs, as detailed below. FA was calculated in numerous ways, including Eq. 1, a modified Lee’s equation, as well as a method proposed by Toeda (Toeda and Kurane, 1991; Lee et al., 1995). The modified Lee’s equation to calculate FA is

$$FA=\left({AS}_{t0}-{AS}_{t1}\right)-\left({AC}_{t0}-{AC}_{t1}\right)$$ (2)

where AS represents the sample absorbance, AC represents the control absorbance, t0 is the time immediately after sample homogenization and t1 is a specific time after homogenization (Lee et al., 1995; Marobhe et al., 2007). Toeda’s method is

$$FA=\frac{1}{A}-\frac{1}{B}$$ (3)

where A and B represent the sample and reference optical densities, respectively. (Toeda and Kurane, 1991) These results have been converted to percentages when necessary, to allow for more meaningful comparisons with results based on Eq. 1.

Numerous plant-derived bioflocculants showed very strong FA. Mallow mucilage had an optimal FA range of 96.3%–97.4% in synthetic water, which contained 100 mg/L kaolin and 10 mg/L humic acid. This was achieved using 12–26 mg/L of mucilage at pH 7 (Anastasakis et al., 2009). Okra mucilage had an optimal FA range of 93–97.3% under the same conditions but required one-half to one-fifth the dosage (Anastasakis et al., 2009). Widely available pectin was found to have an FA of ∼94% when 12 mg/L was combined with clay and 0.2 mM Al^3+^ or Fe^3+^ at pH 3.0 (Yokoi et al., 2002).

### 2.5 Chemically modified bioflocculants

For the purposes of this review, chemically modified bioflocculants refer to two types of materials: 1) Bioflocculants hybridized with conventional organic flocculants, and 2) Bioflocculants chemically treated to polymerize them without the addition of any other materials. These hybrid materials attempt to combine the safety and biodegradability of bioflocculants with the performance and increased solubility of conventional flocculants. Hybridization can bestow significant cationic charge to bioflocculants *via* quaternary ammonium groups, like 3-chloro-2-hydroxypropyl trimethyl ammonium chloride (Lu et al., 2011). This hybrid approach is not without its drawbacks, given the increased processing costs associated with chemical modification and the potential decrease in environmental safety with the inclusion of potentially harmful polymers like polyacrylamide.

All of the reviewed studies (Table 5) utilized polysaccharides as their base material due to the copious reactive sites available. Clay solutions utilized were of lower concentration than the *Bacillus*, *Pseudomonas,* and wastewater studies and were more in line with the plant-based bioflocculation tests, with some studies focusing on NTU-based analysis (Lu et al., 2011). Kaolin clay was not the only type of clay employed, with one study using oppositely charged hematite as well as kaolin in order to demonstrate the importance of chemically modified bioflocculant charge on FA (Li et al., 2015). In addition to examining the clay flocculation performance of chemically modified bioflocculants, some of the articles attempt to optimize the material FA by varying graft ratios and degrees of substitution (Ghimici et al., 2010; Lu et al., 2011; Li et al., 2015).

**TABLE 5:** Chemically modified bioflocculants and their flocculation performance.

Bioflocculant-synthetic polymer hybrids	Bioflocculant composition	Molecular weight (g/mol)	Synthetic polymer chemistry	Synthetic polymer charge	Synthetic polymer-bioflocculant linkage	Degree of substitution	Graft ratio	Measurement of flocculating activity	Mechanism	Optimal efficiency	Effective flocculation additives	Effective (≥80%) temperature range (°C) (max)	Effective (≥80%) pH range (max)	Effective (≥80%) concentration (max)	Optimal flocculating activity	References
*Phyllostachys heterocycla* bamboo pulp cellulose-graft-polyacrylamide	PS (glucose)		Polyacrylamide	Uncharged	Free-radical graft copolymerization	1.31	43.8%	0.04 g bioflocculant, 0.05 g CaCl_2_, 500 mL-0.2 g/L kaolin	Bridging	98%	Ca^2+^		1–7, (7)	80 mg/L	80 mg/L bioflocculant, 100 mg/L CaCl_2_, pH 7	Liu et al. (2014b)
*Phyllostachys heterocycla* bamboo pulp polymerized into dicarboxyl cellulose flocculants	PS (cellulose)			Anionic				Bioflocculant, 0.3 g/L CaCl_2_, 100 mL-0.5 g/L kaolin pH 7	Charge neutralization	∼96%	300 mg/L CaCl_2_		4–10	20–100 mg/L, (40)	carboxylate content 1.24 mmol/g, pH 7, 300 mg/L CaCl_2_, 40 mg/L DCC	Zhu et al. (2015)
Starch + CTA, CMS-CTA-P	PS (amylose + amylopectin)		Poly (3-Chloro-2- hydroxypropyl) trimethylammonium chloride	Cationic	Carboxymethyl starch etherification	43.2% CTA, 20.3% carboxymethyl		0.1 wt% polymer stock solution, 1.0 g/L kaolin or hematite	Charge neutralization, Patching	97.7% (kaolin)			kaolin: 4–11	0.5–5.0 mg/L, (0.8) (kaolin)	kaolin, 0.8 mg/L bioflocculant, pH 11	Li et al. (2015)
Starch + monochloroacetic acid, CMS-CTA-N	PS (amylose + amylopectin)		Poly (3-Chloro-2- hydroxypropyl) trimethylammonium chloride	Anionic	Carboxymethyl starch etherification	27.4% CTA, 62.3% carboxymethyl		0.1 wt% polymer stock solution, 1.0 g/L kaolin or hematite	Charge neutralization, Patching	98.6% (hematite)			hematite: 4–7	1.5–5.0 mg/L, (3.0) (hematite)	hematite, 3 mg/L bioflocculant, pH 7	Li et al. (2015)
(3-acrylamidopropyl)-trimethylammonium chloride-pullulan	PS (maltotriose)	2.0E + 05	Poly (3-acrylamidopropyl) trimethylammonium chloride	Cationic	Graft copolymerization		29.1–52.7%	1 g/L 47% kaolin, 22% montmorillonite, 31% quartz, pH 4.5	Neutralization, Bridging	∼98%		RT		2–14 g/L, (4)	parent solution concentration 1 g/L, 4 g/L [final], 40.9% graft ratio	Ghimici et al. (2010)
Chitosan-3-chloro-2-hydroxypropyl trimethyl ammonium chloride-graft-polyacrylamide	PS (glucosamine, N-acetyl-glucosamine)	8.34E + 05	Polyacrylamide		Graft polymerization	28.2–44.7% CTA	286% acrylamide	0.01–3.0 mg/L bioflocculant, 10–90 NTU kaolin pH 7.0	Bridging, Neutralization	97.5%		5–45	1–9	0.01–3.0 mg/L, (0.1)	44.7% CTA DS, 25°C, 0.10 mg/L, pH 7.0	Lu et al. (2011)
Fenugreek gum grafted with PAM	42.68% Carbon, 8.083% Hydrogen, 33.393% Oxygen	7.75E + 06	Polyacrylamide and ceric ammonium nitrate		Graft copolymerization		1,129.15%	1 ppm bioflocculant, 0.25% kaolin		92.05%		10, 25, 40, 55 (25)	2–10, (4)	1 ppm	1 ppm bioflocculant	Mishra and KunduSynthesis, (2019)
Octopus-like lignin-grafted cationic polyacrylamide		3.27E + 04	Polyacrylamide	Cationic	Free radical polymerization			4.0 mg/L–4.5 mg/L bioflocculant, 10 mg/L PAC coagulant	Charge neutralization, Bridging	92%	10 mg/L PAC		5–9	4.0 mg/L	4.0 mg/L	Chen et al. (2020)

PS, polysaccharide; RT, room temperature.

Bamboo pulp cellulose-grafted polyacrylamide was found to have a very high FA of 98%. Suspension was dosed with 0.04 g of bioflocculant and 0.05 g CaCl_2_ at pH 7.0 (Liu et al., 2014b). When (3-acrylamidopropyl)-trimethylammonium chloride and pullulan were grafted, ∼98% FA was achieved at the highest graft ratio (Ghimici et al., 2010). Artificially polymerized bamboo pulp cellulose had an FA of ∼96% at 40 mg/L DCC with 300 mg/L of CaCl_2_ at pH 7 (Zhu et al., 2015).

### 2.6 Soil bacteria and fungi

*Bacillus* bacteria are not the only soil bacteria known to produce effective bioflocculants. There are numerous other bacterial genera, as well as some fungi, currently being studied. This diversity in the source of bioflocculant-producing microorganisms will naturally lead to a greater variety of bioflocculant molecules, production conditions, and ideal flocculating environments suitable for a range of industrial processes. Another bioflocculation study with broad industrial potential involved supplementing *Agrobacterium tumefaciens* cultures with a quorum sensing signal molecule to directly induce greater bioflocculant production without increasing the bacterial population density (Yang et al., 2016). This unusual but effective approach could be broadly applicable to various large-scale fermentation processes to increase yields and drive down costs.

The majority of the bioflocculants observed for these groups were primarily polysaccharides (Table 6). Only one bioflocculant composed of >50% protein, produced by an *Arthrobacter*, was found (Mabinya et al., 2012). Unlike the majority of the plant-derived bioflocculation tests, kaolin test solutions were of higher concentration, in the standard range of 4–5 g/L. The effective and optimal pH ranges for bioflocculants from soil bacteria and fungi tended to be acidic, in the range of 5-7.

**TABLE 6:** Soil bacteria and fungi and their flocculation performance.

Bacterium (Gram stain)	Bioflocculant composition	Molecular weight (Da)	Measurement of flocculating activity	Mechanism	Optimal efficiency (%)	Effective flocculation additives	Effective (≥80%) temperature range (°C) (max)	Effective (≥80%) pH range (max)	Effective (≥80%) concentration (max)	Condition for optimal flocculating activity	References
*Cellulosimicrobium cellulans* L804 (+)	68.6% PS, 28.0% P	2.29E + 05	Various quantities of bioflocculant added to 5 g/L kaolin	Bridging, Charge neutralization	92.68	Ca^2+^, Mg^2+^	1–70, (35)	2–13, (5)	2.0–22.0 mg/L, (12.0)	Corn stover carbon source, 48-h culture, 12.0 mg/L bioflocculant, 35°C pH 5.0	Liu et al. (2015b)
*Aspergillus niger* A18)	PS		0.1 mL bioflocculant, 100 mL-5 g/L kaolin		95.26		30–100, (30)	5–8, (6)	1 mL/L	1 mL/L bioflocculant broth, 30°C, pH 6.0	Pu et al. (2018)
*Klebsiella mobilus (-)*	PS (NS)		0.2 mL supernatant, 1 mL 1 mg/L CaCl_2_, 100 mL-5 g/L kaolin pH 7		95.4	Ca^2+^	30	3–10, (6)		5% inoculum, pH 6.0, 30°C, 10 μg/L CaCl_2_	Wang et al. (2007)
*Paenibacillus elgii* B69	100% PS (1:0.53:1.15:0.46 glucose, glucuronic acid, xylose, mannose)	3.5E + 06	0.5 mL-1% bioflocculant, 0.5 mL-1% (w/v) CaCl_2_ 10 mL-4 g/L kaolin		87			3–11		0.05% bioflocculant	Li et al. (2013)
*Serratia ficaria (-)*	NS	3.13E + 05	≤0.2 mL cell supernatant, 2 mL-1% CaCl_2_, 100 mL-5 g/L kaolin pH 7	Bridging, Charge neutralization	97.15	Na^+^, Al^3+^, Fe^3+^, Ca^2+^, Mg^2+^	<50	5–7	1.0–5.0 mL/L, (4.0)	4.0 mL/L bioflocculant, 20 mL/L Ca^2+^	Gong et al. (2008)
*Rhizopus* sp. M9 and M17	PS		Various amounts of fermentation broth in 100 mL-5 g/L kaolin	Bridging	95.46		<100	5.5		0.2 mL/L broth, 3:2 M9:M17 ratio	Pu et al. (2014)
*Agrobacterium tumefaciens* strain F2 (-)	93.8% PS		10 mL of crude bioflocculant. 1.5 mL-10% CaCl_2_, 1L-5 g/L kaolin, pH 7.5		89.29	N-hexanoyl-homoserine lactone	30	7	570–640 mg/L	Fermentation with 0.4 μM N-hexanoyl-homoserine lactone, 30°C, pH 7.0, 570–640 mg/L bioflocculant	Yang et al. (2016)
*Arthrobacter* sp. Raats (+)	56% P, 25% C		0.1 mL culture supernatant, 0.25 mL-1% CaCl_2_, 9 mL-4 g/L kaolin pH 7	Neutralization	87.5	Ca^2+^		7		120 h of cell growth	Mabinya et al. (2012)

PS, polysaccharide; P, protein; NS, neutral sugar; C, carbohydrate.

Soil bacteria and fungi yielded some very high performing bioflocculants. *Serratia ficaria* produced a bioflocculant with an optimal FA of 97.15% when cultivated after 72 h with lactose as the carbon source (Gong et al., 2008). A coculture of two strains of Rhizopus sp., M9 and M17, produced a bioflocculant having an optimal FA of 95.46% when dosed with 0.2 mL fermentation broth/L kaolin suspension (Pu et al., 2014). *Klebsiella mobilus* produced a bioflocculant with a peak FA of 95.4% with an initial inoculum volume of 5% was used and the flocculation was carried out at 30°C, pH 6.0 in the presence of 10 μg/L CaCl_2_ (Wang et al., 2007).

### 2.7 Aquatic bacteria and algae

Novel bioflocculant-producing microorganisms are not limited to those found in soil. Numerous studies have identified aquatic bacteria and algae capable of producing bioflocculants with considerable flocculating activity. The significant variations in the natural environment of aquatic bacteria compared to those found in soil further broaden the diversity of microorganisms and molecules capable of efficient bioflocculation. The use of algae for bioflocculant production, demonstrated using *Gyrodinium impudicum*, exemplifies the benefits of diversification as there may be economic benefits by simultaneously using the algae for the production of bioflocculants and algae-related food, oil or pigments (Xia et al., 2018). The inclusion of two *Halmonas* species also exemplifies the benefits of diversification as these deep-sea bacteria are low temperature and salt tolerant, meaning a wider range of culture and effective bioflocculating conditions can be considered, avoiding potential desalinization and reducing the demands for fresh water (He et al., 2010; Okaiyeto et al., 2013).

All of the bioflocculants produced by aquatic bacteria or algae were primarily polysaccharides, with the algal bioflocculant being highly sulfated (Table 7): a result not seen in any of the other studies reviewed herein (Yim et al., 2007). Kaolin (4–5 g/L) was the test solution for all of these studies, allowing for a direct comparison with the majority of the other results in this review. Cations were necessary in 60% of the studies, with the remaining 40% showing enhanced flocculation in the presence of cations. The effective pH range for flocculation ranged from 4-8.

**TABLE 7:** Aquatic bacteria and algae and their flocculation performance.

Bacterium (Gram stain)	Bioflocculant composition	Molecular weight (Da)	Measurement of flocculating activity	Mechanism	Optimal efficiency (%)	Effective flocculation additives	Effective (≥80%) temperature range (°C) (max)	Effective (≥80%) pH range (max)	Effective (≥80%) concentration (max)	Condition for optimal flocculating activity	References
*Klebsiella variicola* BF1 (-)	83.1% C, 10.6% P		0.1 mL bioflocculant, 1 mL-10 g/L CaCl_2_, 9 mL-5 g/L kaolin	Adhesion	97.6	Ca^2+^, Mg^2+^, Fe^2+^, max 2.5 g/L CaCl_2_	30	6.5	5–50 mg/L, (12.8)	12.8 mg/L BP-1, 2.5 g/L CaCl_2_	Nguyen et al. (2019)
*Halomonas* sp. Okoh (-) and *Micrococcus* sp. Leo (-)	62.3% S, 25.7% UA, 4.73% P		2 mL bioflocculant, 3 mL 1% CaCl_2_, 100 mL-4 g/L kaolin	Neutralization	86	Al^3+^		8		1.9 mg/L bioflocculant. pH 8	Okaiyeto et al. (2013)
*Acinetobacter junii* BB1A (-)	83.56% PS (73.21% NS, 10.12% UA, 0.23% AS), 12.36% P	2.0E+05	0.1 mL bioflocculant, 5 g/L kaolin	Neutralization, Bridging	97	30 mg/L bioflocc w/o cation OR 0.7 mM CaCl_2_, Zn^2+^, Cu^2+^, Cd^2+^, Mn^2+^, Co^2+^, Mg^2+^, and Ni^2+^	20	4–10 w/CaCl_2_, (5). 4–5 w/out CaCl_2_, (5)	30–50 mg/L, (30 w/o CaCl_2_)	pH 7 and 0.7 mM CaCl_2_, or 30 mg/L bioflocculant, pH 5.0, 20°C	Yadav et al. (2012)
*Gyrodinium impudicum* KG03	Galactose (3.0% UA, 10.3% Sulfate) 2.58% P	1.87E+06	0.4 mL bioflocculant, 0.4 mL 5% CaCl_2_, 19.2 mL-5 g/L kaolin	Bridging	93.6	90.5% w/o cation, 93.6% with Ca^2+^	4–90	3–6, (4)	0.5–2.5 mg/L, (1.0)	1.0 mg/L bioflocculant, 0.1% CaCl_2_	Yim et al. (2007)
*Halomonas* sp. V3a’ (-)	PS (20.6% NS, 7.6% UA, 1.6% AS)	5.90E+05	0.4 mL bioflocculant, 1.0 mL CaCl_2_,100 mL-5 g/L kaolin	Bridging	96.9	Ca^2+^	5–40, (25)	3–11, (7)	1–16 mg/L, (4)	4.0 mg/L bioflocculant, 11.25 mM CaCl_2_, pH 7.0, 25°C	He et al. (2010)

C, carbohydrate; P, protein; S, sugar; UA, uronic acid; PS, polysaccharide; NS, neutral sugar; AS, amino sugar.

*Klebsiella variicola* BF1 produced a bioflocculant having an optimal FA of 97.6% when suspension was dosed with 12.8 mg/L of bioflocculant and 2.5 g/L of CaCl_2_ (Nguyen et al., 2019). *Acinetobacter junii* BB1A produced a bioflocculant having an optimal FA of 97% when suspension was maintained at pH 7 and dosed with 30 mg/L of bioflocculant or 0.7 mM CaCl_2_ (Yadav et al., 2012). *Halomonass* sp. V3a’ produced a bioflocculant having an optimal FA of 96.9% when suspension was maintained at pH 7 and 25°C with 11.25 mM Ca^2+^ and 4 mg of bioflocculant (He et al., 2010).

## 3 Future directions and conclusion

This review summarizing the different research attempts towards a better bioflocculant of kaolin demonstrates that a wide range of potent bioflocculants and sources have been found, and this distribution of high FA sources and materials limits the opportunity for the formation of a general consensus or direction in the field. These works are still very exploratory, with few studies iterating and improving upon previous works, and/or focusing on kaolin flocculation specifically. In most instances, kaolin solutions are used as a simple screening test to show FA, then the bioflocculants are applied to other sludge or wastewater solutions. This ultimately leaves the field of kaolin bioflocculation underdeveloped, despite the immense quantities of kaolin tailings requiring remediation, including ∼4.8 billion barrels of mature fine tailings from oil sands production (Schindler, 2014; Wang et al., 2014; Liu et al., 2020; Anagnostopoulas et al., 2022). The intention of this review is to highlight some common sources and characteristics of effective kaolin bioflocculants, with a mind towards future technically and economically viable applications of these systems.

Taken as a whole, the studies summarized herein demonstrate that bioflocculants can have significant kaolin FA over a wide range of pH and temperature conditions, alleviating some of the need to bring kaolin solutions to standard pH and/or temperature ranges for efficient flocculation. In fact, optimal FA was observed at pH values ranging from 1–12 and temperatures from 5–80°C. *Bacillus*-derived bioflocculants tended to show optimal FA over the broadest range of pH values including extremely acidic and alkaline conditions, with wastewater and plant-derived bioflocculants also showing some optimal activity under a greater range of alkaline conditions. Most optimal FA conditions were at temperatures ranging from 20–35°C, but both *Bacillus* and wastewater-derived bioflocculant categories contained at least five studies where effective FA (≥80%) was achieved at 0–10°C, with Cassava wastewater biological aerated filter sludge and biological sludge exhibiting optimal FAs of 92.67% and 99.5% at 10°C and 5°C, respectively (Liu et al., 2009; Sun et al., 2012). Seven *Bacillus*-derived bioflocculants were shown to have effective FA (≥80%) at temperatures from 60–95°C, with *Bacillus aryabhattai* PSK1 having an optimal FA of 94.56% at 50°C (Abd El-Salam et al., 2017). Multiple wastewater-derived *Klebsiella*, *Rhodococcus* and *Enterobacter* bioflocculants also showed effective FAs at high temperatures, with *Enterobacter cloacae* WD7 having optimal FA of ≥91% at 50°C and *Klebsiella variicola* B16 peaking at 96.18% FA at 60°C (Prasertsan et al., 2006; Xia et al., 2018).

Of the 73 studies which quantified the composition of the bioflocculants, 53 were primarily polysaccharide, 10 were protein, and 10 were composed of significant proportions of both polysaccharide and protein. The compositions of the polysaccharide portions of the bioflocculants varied, with a range of proportions of neutral sugars, amino sugars and uronic acids. The uronic acid content in particular shifted considerably, from 1.2–59.3% of the total polysaccharide (Sathiyanarayanan et al., 2013; Chen et al., 2016). The uronic acid content is noteworthy because these are sugar derivatives containing carboxylic acid functional groups which are know to participate in kaolin particle attachment (Xia et al., 2018). Carboxylic acid chemistries also make up the sidechains of glutamic acid and aspartic acid, amino acids that were enriched in most of the protein components of the bioflocculants reviewed herein. Three studies found that polyglutamic acids, proteins composed almost exclusively of glutamic acid, were responsible for kaolin bioflocculation (Shih et al., 2001; Bajaj and Singhal, 2011; Zhao et al., 2013). Carboxylic acid functional groups are replete throughout common synthetic polymer flocculants including polyacrylamide, and the structural and charge similarities of acrylamide monomers and uronic acid as well as the amino acids glutamic acid and aspartic acid cannot be overlooked as these chemical similarities may be one of the main driving forces of bioflocculant efficacy (Liu et al., 2020).

While these studies demonstrate the range of bacteria capable of producing effective kaolin bioflocculants, the limitations of these studies must also be addressed. Complete treatment of kaolin tailings requires the dewatering of the settled material, a topic which was only examined in 7 of the manuscripts, primarily regarding the dewatering of sludge rather than kaolin. Dewatering releases bound water, reduces the tailings volume and improves the mechanical strength of the clay, factors necessary for the proper disposal of these materials. Dewatering efficiency can be impacted by the choice of flocculant with, for instance, aluminum-polyacrylamide hybrid polymers leading to rapid dewatering (Wang et al., 2014). The kaolin concentrations used to assess bioflocculation were limited almost exclusively to 5 g/L or less, conditions forty to sixty times more dilute than oil sands tailings. As such, to accurately gauge bioflocculant FA for applications such as treating oil sands tailings, further testing will be necessary as the composition of oil sands tailings is much more complex than just kaolin. Another line of inquiry relevant for successful industrial deployment of bioflocculants is the *in situ* stability of the flocculating molecules. While some studies did examine bioflocculant pH and temperature stability, the results only demonstrate the retention of FA over a short period of time. Bioflocculant stability *in situ* over longer periods of time would more accurately reflect real-world applications, and may impact the overall FA and dewatering outcomes. Safety is another limitation that has not been fully addressed. While bioflocculants can be safer alternatives to conventional inorganic and organic polymeric flocculants, the hazards arising from the microorganisms producing the bioflocculants must be kept in mind. For instance, the use of *Enterobacter* and *Pseudomonas aeruginosa* for bioflocculant production poses a serious hazard due to their pathogenicity in humans. Furthermore, large scale growth of *Gyrodinium* or similar algae blooms can lead to toxic conditions towards marine and mammalian life forms, including humans, in addition to potentially inhibiting flocculation (Sano et al., 2011).

Even as studies like those reviewed herein advance the scientific understanding of the effective compositions for bioflocculants, optimization of bioflocculant production, range of bioflocculant-producing microorganisms, and FA, the widespread deployment of bioflocculants needs to overcome the economic hurdles accompanying their use. The vast volumes of kaolin tailings requiring treatment, ∼4.8 billion barrels worth from oil sands production alone dictates that any reasonable treatment solution must be economically, as well as technically, viable. Even the addition of a small amount of CaCl_2_ represents a significant cost when scaled to industrial proportions, and in the case of recycled water applications can reduce the efficiency of bitumen extraction. Every effort must be made to drive bioflocculant production costs down. Some of the reviewed studies omitted bioflocculant purification while retaining considerable FA, a strategy that minimizes sample preparation and the costs associated with it. Another potential option not well explored in the literature insofar would be to culture the bioflocculant-producing microorganisms in the tailings they are meant to treat. This would eliminate and transportation costs and the need for a large-scale offsite culturing facility, and any nutrients found in the tailings would offset at least a portion of the costs of the culture broth. While the current studies optimize bioflocculant production and performance and begin to consider minimizing purification and nutrient requirements, the use of more advanced bioengineering techniques common to fields like biopharmaceutical production should also be considered to further enhance bioflocculant production and performance to minimize costs. Two such strategies, each seen in only one bioflocculation study, the enhanced induction of exopolysaccharide bioflocculant production using quorum sensing-associated *N*-acyl homoserine lactones and the directed evolution of bioflocculant-producing microorganisms, have been reported to enhance bioflocculant production and performance (He et al., 2010; Yang et al., 2016). These are powerful methods which must be applied to bioflocculation further. Metabolic engineering has been shown to enhance exopolysaccharide and polyglutamic acid production for other applications, and must be employed to enhance bioflocculant production (Levander et al., 2002; Henry and Durocher, 2011; Feng et al., 2015; Cai et al., 2018). The bioflocculant design principles gleaned from this review could also be used for the intelligent design and recombinant production of repetitive proteins for use as bioflocculants (Pickens et al., 2011; Bahniuk et al., 2016). Biopanning strategies are well suited for high-throughput screening of bioflocculant-producing microbes and their mutants produced using any of the preceding bioengineering methods, using adhesion to kaolin particles as the separation method (Sano et al., 2011). Naturally, the costs associated with these methods would need to scale appropriately with the scale of the bioflocculant production and treatment, and these methods have the potential to scale very well with cost, as, unlike direct media supplementation, mutations and recombinant plasmids tend to be naturally replicated and maintained in the cell culture as it propagates and scales.

The production of bioflocculants may face significant barriers to their application on an industrial scale. Perhaps the first major barrier to the application of bioflocculants revolves around safety; systems that use microorganisms as bioflocculants need to be designed so as to prevent unwanted release, in tandem with microbe selection to reduce any potentially harmful effects of release. These issues must include the input from a broad group of experts, including governmental safety leaders, biochemists, and application engineers to ensure public safety is maintained. Moreover, should the bioflocculant need to be produced by the microorganisms and purified, without properly assessing production yields the technology would be uneconomical. Although there are potential risks to the application of microorganisms as bioflocculants, one should remember that there may be significant benefits that could be realized as well. For example, if a microorganism can be a good bioflocculant but also fix carbon within the tailings environment a significant carbon capture strategy could be developed to reduce greenhouse gases. Moreover, the economic burden of deploying microorganisms could be offset through production of a high-value commodity, and using the ‘waste’ microorganisms as the bioflocculant. After 25 years of research it is evident that we have only started to apply biochemical techniques to the significant environmental liabilities that kaolin tailings present.

## Abbreviations

FA, flocculating activity; NTUs, nephelometric turbidity units.
